# Preliminary estimates of the abundance and fidelity of dolphins associating with a demersal trawl fishery

**DOI:** 10.1038/s41598-017-05189-0

**Published:** 2017-07-10

**Authors:** Simon J. Allen, Kenneth H. Pollock, Phil J. Bouchet, Halina T. Kobryn, Deirdre B. McElligott, Krista E. Nicholson, Joshua N. Smith, Neil R. Loneragan

**Affiliations:** 1 School of Veterinary and Life Sciences, Murdoch University, Murdoch, Western Australia 6150 Australia; 2 School of Biological Sciences and Oceans Institute, University of Western Australia, Crawley, Western Australia 6009 Australia; 3 Evolutionary Genetics Group, Department of Anthropology, University of Zurich, Winterthurerstrasse 190, 8057 Zurich, Switzerland; 4 Department of Applied Ecology, North Carolina State University, Raleigh, North Carolina 27695-7617 United States of America; 5 Daithi O’Murchu Marine Research Station, Gearhies, Bantry, Co. Cork Ireland

## Abstract

The incidental capture of wildlife in fishing gear presents a global conservation challenge. As a baseline to inform assessments of the impact of bycatch on bottlenose dolphins (*Tursiops truncatus*) interacting with an Australian trawl fishery, we conducted an aerial survey to estimate dolphin abundance across the fishery. Concurrently, we carried out boat-based dolphin photo-identification to assess short-term fidelity to foraging around trawlers, and used photographic and genetic data to infer longer-term fidelity to the fishery. We estimated abundance at ≈ 2,300 dolphins (95% CI = 1,247–4,214) over the ≈ 25,880-km^2^ fishery. Mark-recapture estimates yielded 226 (SE = 38.5) dolphins associating with one trawler and some individuals photographed up to seven times over 12 capture periods. Moreover, photographic and genetic re-sampling over three years confirmed that some individuals show long-term fidelity to trawler-associated foraging. Our study presents the first abundance estimate for any Australian pelagic dolphin community and documents individuals associating with trawlers over days, months and years. Without trend data or correction factors for dolphin availability, the impact of bycatch on this dolphin population’s conservation status remains unknown. These results should be taken into account by management agencies assessing the impact of fisheries-related mortality on this protected species.

## Introduction

As both the human population and our demand for seafood grow, the incidental capture, or bycatch, of non-target species in fisheries continues to present a global conservation challenge^1–3^. Bycatch in fisheries is widely recognized as the most pressing threat to the persistence of many populations of marine megafauna^4, 5^. For example, entanglement in fishing gear contributed to the extinction of the Yangtze River dolphin (*Lipotes vexillifer*)^6^. Fishing’s direct (bycatch or targeted hunting) and indirect impacts (habitat modification and prey depletion) are also implicated in declines that may be irreversible in marine megafauna including: common dolphins (*Delphinus delphis*) in the Mediterranean Sea^7^; finless porpoises (*Neophocaena asiaeorientalis asiaeorientalis*) in the Yangtze estuary^8^; the endemic sea lions (*Phocarctos hookeri* and *Neophoca cinerea*) of New Zealand^9^ and Australia^10^; shark populations globally^11^; and vaquitas (*Phocoena sinus*) in the Gulf of California^12^. In addition to exacerbating global biodiversity loss, unsustainable fishing and the removal of populations of megafauna, apex predators in particular, may also have cascading effects on the structure of communities and ecosystem function^13–16^.

Resolving megafauna bycatch is challenging as it is often poorly understood, inadequately documented, and it varies considerably between fishery types and the species subject to capture^17, 18^. There has also been a lack of coordination and common purpose between the agencies with the mandate to manage fisheries and those charged with the conservation and management of marine megafauna^17, 19^. Furthermore, the life history traits of marine megafauna (slow growth, late maturation and low reproductive rates) render many species vulnerable to population-level impacts. Ensuring compliance with policies designed to minimise bycatch is also particularly difficult when the economic incentives from fishing discourage a genuine commitment to conservation^1, 5^. Policy controls implemented in order to curb marine megafauna bycatch in commercial fisheries include the *Marine Mammal Protection Act* (1972) in the United States and New Zealand’s *Marine Mammal Protection Act* (1978). Under such legislation, the maximum level of fishing-related mortality allowed for each impacted marine mammal population, stock or management unit is calculated using concepts such as Potential Biological Removal or the Maximum Allowable Level of Fishing-Related Mortality^9, 20^. These initiatives have met with varying degrees of success, although recent reviews illustrate that baseline information on population trends, critical to assessing and managing bycatch, is lacking for the majority of marine mammal species^21, 22^.

A number of different commercial fisheries operate within Australia’s expansive Economic Exclusion Zone (the world’s third largest), overlapping with a diverse assemblage of cetaceans. The greatest proportion of cetacean bycatch in Australian waters results from gill netting, purse seining and trawling^23–25^, as it does globally^1^. All cetaceans are protected under Australia’s *Environment Protection and Biodiversity Conservation Act* (1999), a provision of which stipulates that a commercial fishery should receive accreditation only if it does not, or is not likely to, adversely affect the conservation status of a cetacean, or a population of cetaceans. Although cetacean bycatch rates are likely to have been decreasing in Australian fisheries over the last few decades, even abundance data do not exist for most populations, particularly for delphinids^22^. Attempts have been made to estimate cetacean bycatch levels from observer data or implement trials for bycatch reduction around Australia^23, 24, 26^ but without abundance estimates, trend data, or an understanding of what proportion of the impacted populations is affected, assessing the level of risk to the viability of cetacean populations subject to fisheries bycatch is not possible^20, 27^.

**Figure 1:**
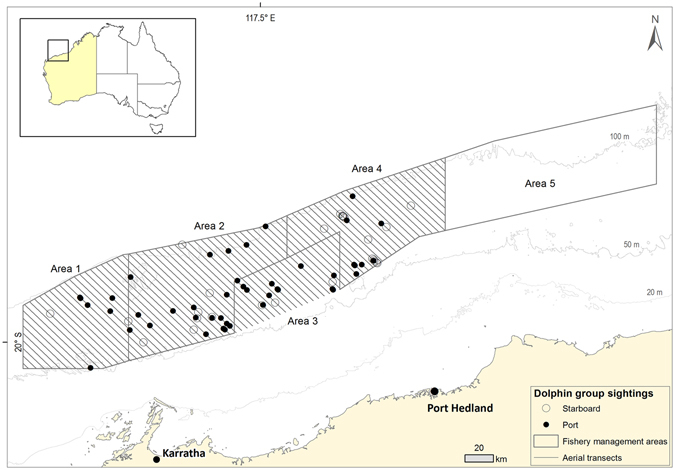
Aerial tracks surveyed in April 2011 across the Pilbara Trawl Fishery, north-western Australia; Fishery management areas 1, 2, 3 and 4 were surveyed (trawling is not permitted in area 3; area 5 was not surveyed); Circles indicate dolphin sightings (● = port observer/s, ○ = starboard observer); 20 m, 50 m and 100 m depth contours are also indicated. This figure was generated in ArcGIS v10.1.

Common bottlenose dolphins (*Tursiops truncatus*, ‘bottlenose dolphins’ hereafter) are well known globally^28^, but considered ‘data deficient’ around Australia, where they tend to occur in pelagic habitats, more distant from the coast and human population centres than the closely related Indo-Pacific bottlenose dolphins (*T. aduncus*)^22, 29^. No population estimates for *T. truncatus* exist in Australian waters, and our limited knowledge from north-western Australia is a result of relatively recent research due to their bycatch in the Pilbara Fish Trawl Interim Managed Fishery (‘Pilbara Trawl Fishery’, or ‘PTF’, hereafter - Fig. 1)^25, 30^. Bycatch rates were estimated at approximately 50 dolphins year^−1^ from independent observer data collected over a six-year period (2003–2009), more than double the rate reported by skippers^25^. Furthermore, some dolphins are caught and then expelled from escape hatches in bycatch reduction devices before winch up^30, 31^. Thus, mortality rates are under-estimated in the PTF, because of both under-reported bycatch and the unobserved loss of dead or moribund dolphins during trawling^25^. In 2012, a six-month trial of an electronic (video cameras) observer system was conducted to compare the efficacy of different bycatch reduction devices and quantify megafauna bycatch, but this was not verified against independent (human) observer data^32^. Under-reporting in fisheries statistics, especially the bycatch of protected species, is a common phenomenon elsewhere around Australia^33^ and globally^34^.

Here, we combined aerial survey of the fishery, boat-based dolphin photo-identification around one trawler (of three operating at the time of the study), and photographic and genetic data collected opportunistically around all three trawlers operating in this fishery to: (*i*) estimate the abundance of bottlenose dolphins across the PTF; (*ii*) estimate the number and assess short-term fidelity of dolphins foraging behind one trawler over periods of days to weeks; and (*iii*) infer fidelity to foraging around trawlers over months to years. This research was carried out to provide preliminary estimates of the abundance and fidelity of dolphins associating with the PTF, and a baseline from which to assess whether ongoing dolphin bycatch is “acceptable”^35^ or poses “negligible risk” to the population^32^ in the future.

## Results

### Aerial survey of the Pilbara Trawl Fishery to assess dolphin abundance

Eleven flights were carried out between the 11^th^ and the 22^nd^ April 2011, across four of the five Pilbara Trawl Fishery management areas (Fig. 1; Methods). A series of 81 transects, oriented NW-SE, broadly perpendicular to the coastline and spaced 2 nm apart (covering a combined linear distance of 4,937.4 km) were surveyed by a team of three experienced observers at an altitude of 500 feet and a cruising speed of 100 knots. As the speed of the aircraft is much higher than the swimming speed of the animals, counts from the aircraft effectively provided a snapshot of animal abundance in the area at the time of the survey. The number of weather-dependent flights carried out across the survey period ranged from 0–2 per day, the average flight time was 3 h 35 min and the average transect length across the fishery was 61 km.

Personnel for all surveys included two pilots, a data recorder, one starboard observer, and two visually and acoustically independent portside observers. The observers and data recorder were linked via a separate intercom system, and data were logged with a time code to a digital tape recorder. Observers measured vertical angles from the plane to each dolphin group sighted using hand-held clinometers. Perpendicular distances were calculated using trigonometry, based on known heights and vertical angles. In addition to perpendicular distance, the following variables were recorded: (1) Group size (S); (2) Fatigue (F); (3) Time of day (T); (4) Beaufort sea state (B); (5) Calves (CA); (6) Cloud cover (CC); (7) Glare intensity (G); (8) Glare angle (GA) (see Methods for further details). As Mark-Recapture Distance Sampling (MRDS) analysis can capture some degree of perception bias, we focus on the results from the dual platform (fore and aft), port side only.

A total of 82 non-trawler-associated bottlenose dolphin clusters were counted during the aerial survey, ranging in size from 1 to 30 individuals (mean ± 1 SE = 5.0 ± 0.6, Fig. 1). Three clusters of trawler-associated dolphins were also counted during the aerial survey (see below). As these aggregations of dolphins could have unduly inflated the modelled abundance estimates, they were excluded from the MRDS analyses. After truncation and data filtering of the non-trawler associated dolphin clusters (see Supplementary Information), a total of 61 separate dolphin groups were recorded over all surveys. Of these, 36 sightings were made by the dual observer team on the port side of the plane and retained for MRDS analysis (Table 1).

**Table 1:** Summary statistics from the Mark-Recapture Distance Sampling analysis of dolphin data: n_1,2_ is the number of detections made by observers 1 and 2, respectively; n_3_ is the number of duplicate sightings; n_._ is the total number seen, calculated as n_1_ + n_2_ – n_3_; and the p_s_ are the conditional detection probabilities.

Distance (m)	n_1_	n_2_	n_3_	n_._	p_1|2_	p_2|1_
0–75	7	10	7	10	0.70	1.00
75–150	12	7	6	13	0.85	0.50
150–225	5	4	4	5	1.00	0.80
225–300	1	2	0	3	0.00	0.00
300–375	2	2	1	3	0.50	0.50
375–400	1	1	0	2	0.00	0.00
Totals	28	26	18	36		

Results are broken down into 75 m distance bins away from the transect line.

A total of 38 MRDS models, ranging in complexity from single main factors to multiple factors with interaction terms, were fitted to the aerial survey data (Supplementary Table S1). The best model (minimizing Akaike’s information criterion, AIC) fitted glare angle, time of day and fatigue, and estimated an abundance of 1,551 individuals for the four managed areas surveyed (95% confidence interval = 822–2,929, Table 2; Supplementary Table S1). Note that, although this was our final choice, a number of other candidate models had AIC values within three units of this model and gave estimates of abundance ranging from 1,430 to 1,989 (Table 2). The Multiple Covariate Distance Sampling (MCDS) model estimates, based on all sightings (from both port and starboard observers) and the same truncation and filtering conditions as used in the MRDS analysis, were similar in magnitude, but are not reported here.

**Table 2:** Point Independence (PI) and Full Independence (FI) model details and selection results for the Mark-Recapture Distance Sampling analysis of bottlenose dolphin dual observer data; The model for the conventional detection function g.

Model	Point Independence						Full Independence						
	g. (y, z)	N	N_low_	N_high_	D	AIC	ΔAIC	ΔAIC	N	N_low_	N_high_	D	AIC
Dist + T + F	S	1,653	1,006	2,716	0.09055	193.2	83.0	1.2	1,484	814	2,705	0.08129	111.4
Dist + T + G	S	1,680	1,017	2,776	0.09205	195.0	84.8	2.9	1,578	818	3,046	0.08647	113.1
Dist + T + GA	S	1,651	1,003	2,716	0.09043	193.5	83.3	2.7	1,430	790	2,587	0.07835	112.9
Dist + T + F + S	S	1,650	1,004	2,711	0.09040	195.2	85.0	2.7	1,473	810	2,679	0.08072	112.9
**Dist + T + F + GA**	S	1,656	1,006	2,728	0.09075	193.7	83.5	**0.0**	**1,551**	**822**	**2,929**	**0.08499**	**110.2**
Dist + T + F + G	S	1,679	1,016	2,776	0.09201	196.3	86.1	2.8	1,633	833	3,203	0.08948	113.0
Dist + T + F + GA + F:T	S	1,657	1,005	2,733	0.09079	195.7	85.5	2.1	1,551	821	2,930	0.08497	112.3
Dist + T + F + S + S:F	S	1,762	972	3,193	0.09654	193.6	83.4	3.0	1,855	646	5,325	0.10163	113.2
Dist + T + F + GA + S + S:F	S	1,768	964	3,243	0.09687	194.3	84.1	2.3	1,989	640	6,182	0.10899	112.5

(y, z) is only relevant in the PI scenario; The best model (selected based on the AIC scores) is shown in bold; along with eight other models that had AIC values within three of the best model; Details for all 38 models are shown in Supplementary table S1; Covariate terms are as follows: Dist = distance, F = fatigue, G = glare intensity, GA = glare angle, S = group size, T = time of day. Colons “:” code for variable interactions; Derived parameters include animal density (D), abundance (N) and 95% CI (N_low_; N_high_).

The area surveyed represents 71% of the total PTF. When scaled by area, the estimate from the MRDS analysis with the lowest AIC gave an abundance estimate of 2,185 (95% CI = 1,158–4,125) dolphins for the entire PTF. Note that this extrapolation assumes that the dolphins are distributed uniformly throughout all managed areas of the PTF. The unsurveyed area of the fishery was that furthest from the fishing ports and, as a consequence, its extremities are subject to the least trawling activity. Since no differences were detected in bycatch rates between management areas^25^, we had no reason to expect marked differences in dolphin abundance or density between management areas.

The three clusters of trawler-associated dolphins (N = 89 individuals, mean group size ± SE = 29.7 ± 13.9) were part of the population within the bounds of the PTF aerial survey area. These numbers were therefore added to the estimated population size of non-trawler associated dolphins, yielding a total abundance estimate across the PTF (including management area 5 and trawler-associated dolphins) of 2,274 (95% CI = 1,247–4,214) dolphins.

### Photo-identification of trawler-associated dolphins to assess short-term fidelity

**Figure 2:**
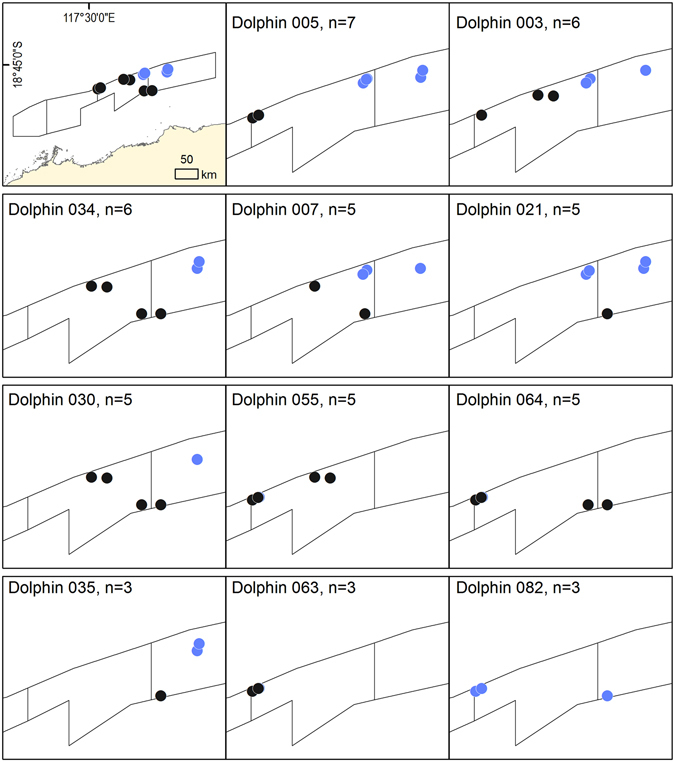
The location of the 12 dedicated photo-identification surveys conducted from one trawler in April 2011 (top left frame) and the photographic captures/recaptures of the eight bottlenose dolphin (*Tursiops truncatus*) individuals sighted five to seven times ( = trip 1; ● = trip 2); Also shown in the bottom panels are the locations of the three individuals (dolphin identification numbers 035, 063, and 082) sighted three times in April 2011 that were matched with opportunistically collected images taken in October and November 2008 (initial locations not shown). This figure was generated in ArcGIS v10.1.

The dedicated photo-identification surveys to assess the numbers and fidelity of dolphins associating with one trawler in April 2011 were concentrated in the central east of the fishery (Fig. 2, top left frame). Approximately 1,400 photographs of dolphins were taken during the 12 photo-identification surveys over 15 days, yielding a catalogue of 136 individually recognisable, trawler-associated dolphins and 251 individual “captures”. Dolphin group sizes ranged from 16 to 46 individuals, with a mean of 28.0 ± 3.0 (n = 12), similar to that from the three aerial survey sightings of trawler-associated dolphin groups (29.7 ± 13.9); and significantly greater than that of non-trawler-associated dolphin groups (5.0 ± 0.6; n = 82; *T*_92_ = 12.1; *P* < 0.0001). Individual sighting frequencies ranged from one to seven in the 12 capture periods, with eight dolphins being photographed between five and seven times (Fig. 2). The abundance estimates for all 12 capture periods around the one (of three) trawlers ranged from 170 ± 8.7 to 210 ± 35.5 marked individuals for the three population models (Table 3).

**Table 3:** Comparison of the estimated population size ($\hat{{\bf{N}}}$), standard error (SE) of the estimate and 95% confidence limits for closed and open models using the graded photo-identification mark-recapture data to estimate the size of the community of marked, trawler-associated dolphins over the two fishing trips (12 samples) on one trawler in the Pilbara Trawl Fishery in April 2011; The three best-fitting population models were: *M*_(t)_ = allowing for variation in capture probability with time; *M*_(th)_ = allowing for both time and individual heterogeneity in capture probability; and Popan = an open population model, assuming closure within sampling days.

Model		$\hat{{\bf{N}}}$	SE($\hat{{\bf{N}}}$)	95% lower	95% upper
*M*_(t)_		170	8.7	157	191
*M*_(th)_		195	17.7	170	241
Popan		210	35.5	166	317

The proportion of distinct individuals in the population was estimated to be 0.93. Using the number of marked animals estimated from the Popan model (Table 3) and scaling to allow for unmarked individuals (see Supplementary Information), the total abundance of dolphins associated with one trawler over the sampling period was 226 ± 38.5 dolphins.

### Opportunistic photographic and genetic sample matching to infer long-term fidelity

Three dolphins that were photo-identified in April 2011 were matched with images of dolphins collected opportunistically in October and November 2008 (Fig. 2, bottom three frames).

**Figure 3:**
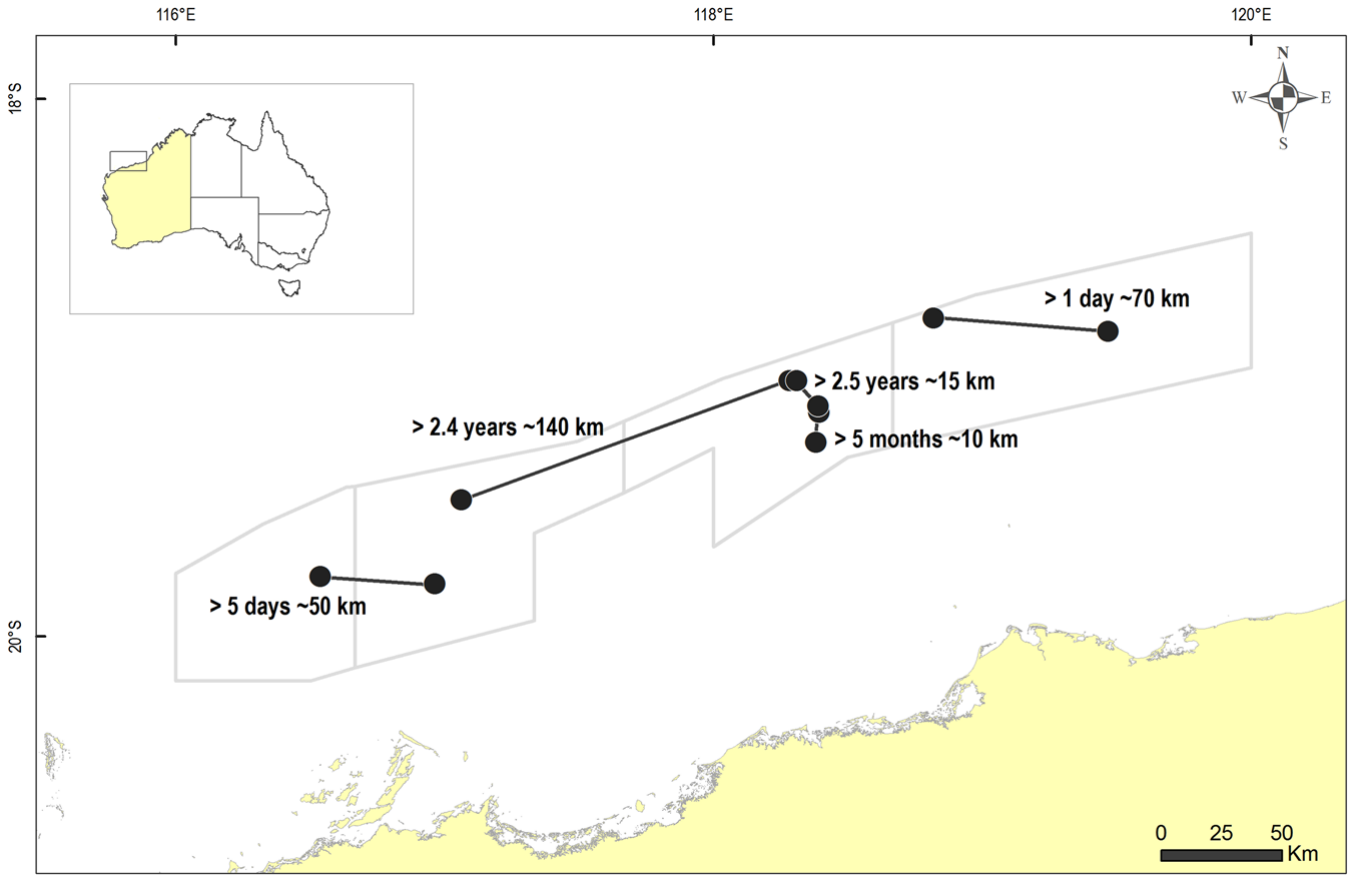
Locations, times and distances between repeat biopsy-sampling events of the same five bottlenose dolphin (*Tursiops truncatus*) individuals (revealed by genetic identity analyses based on microsatellite data) taken at least one day following initial sampling; Lines join circles representing the same individuals (n = 5). This figure was generated in ArcGIS v10.1.

Furthermore, five individual dolphins were biopsy sampled up to 2.5 years after their initial sampling on previous fishing trips between 2008 and 2011. Although repeated sampling of individuals was unintentional, this provided us with the opportunity to infer site fidelity over months and years for at least some individuals. The distances between repeat biopsy-sampling events ranged from 15 km to 140 km and were not related to the time between events (Fig. 3).

## Discussion

This study presents the first abundance estimate for any pelagic delphinid interacting with an Australian fishery, and *a fortiori*, for any pelagic bottlenose dolphin community (*Tursiops truncatus*) in Australian waters. The lack of other such estimates precludes regional comparisons of population sizes in Australasia. Furthermore, there are, as yet, no correction factors for availability bias (dolphins are unavailable for detection, e.g. they are submerged at a depth beyond which they might be observed) based on independent data of surfacing intervals for dolphins in Australian waters. Since our estimate could not be corrected for availability bias, it is likely to be an under-estimate of the number of dolphins in the area at the time. Estimates of ca. 2,000 to 3,000 dolphins in the 25,880 km^2^ fishery area, or ca. 0.1 dolphins/km^2^, are, nevertheless, lower than expected, based on the findings from other regions. These findings bear some comparison, as they too were not corrected for availability bias. Aerial surveys over a similar-sized area (26,700 km^2^) in the Gulf of Mexico, USA, for example, resulted in an estimate of 5,141 bottlenose dolphins (0.19 dolphins/km^2^)^36^, about double the abundance of dolphins estimated in the PTF. Similar numbers of bottlenose dolphins (2,225) to those estimated in the PTF were reported in the Mississippi Sound, USA^37^, but they occupied an area of 2,104 km^2^, <10% of the PTF, indicating a density about ten times greater than in the PTF. Even taking seasonal fluctuations in habitat use into account, the dolphin density in the comparable offshore areas of the Mississippi Sound^37^ were two to three times higher than the density of those in the PTF.

A number of abundance and density estimates have been reported for bottlenose dolphin populations inhabiting various parts of the Mediterranean Sea that are similar in magnitude to the low densities estimated for the PTF^38, 39^. Researchers have attributed the low densities in the Mediterranean Sea to a combination of centuries of overfishing, targeted ‘fishing’ for dolphins of varying magnitudes in some areas, habitat degradation through coastal development and, more recently, increasing commercial and recreational vessel traffic^40–42^. The coastline inshore of the PTF is sparsely populated and remote by comparison to the Mediterranean, and only in the last few decades subject to the pressures of heavily industrialised anthropogenic activity^25, 43^. Incidental fishing-related mortalities therefore appear likely to be the major source of anthropogenic mortality for dolphins in this region.

Although Mark-Recapture Distance Sampling (MRDS) can capture some degree of perception bias (visible dolphins are missed by observers), no correction factors are known for availability bias in this region. The scale of the difference that an availability correction might make to our abundance estimates is difficult to determine, particularly as surfacing behaviour may vary seasonally, ontogenetically and by habitat^44^. As, at least, a loose basis for comparison, however, Slooten *et al*.^45, 46^ reported the average availability of Hector’s and Maui’s dolphins (*Cephalorhynchus hectori* and *C. h. maui*) in New Zealand’s coastal waters as 46% and 56%, respectively. Bottlenose dolphins in the PTF may engage in longer dives (at least when foraging) than Hector’s and Maui’s dolphins, but they occupy consistently clearer waters than the turbid habitat of New Zealand’s endemic species. It remains to be seen whether the average availability determined for these two species, around 0.5, is an apt estimate for bottlenose dolphins in the PTF. Pollock *et al*.^47^ estimated the availability of dugongs (*Dugong dugon*) at a range of depths, turbidities and sea states in Northern Australian coastal waters for application to aerial survey data. Availability probability estimates for these grazers varied between 0.3 and 1.0^47^. The bottlenose dolphins surveyed in the PTF could reasonably be expected to have a higher availability than foraging grazers in turbid waters and sea states ≥ 2. If bottlenose dolphin availability was as low as 0.3, however, our corrected abundance estimate would still remain markedly fewer than 10,000 (our estimate of 2,274/0.3 ≈ 7,580) PTF-associated dolphins.

Whether or not the abundance of bottlenose dolphins in the PTF fluctuates seasonally is unknown, but no temporal variations were detected in the rates of dolphin bycatch among seasons between 2003 and 2009^25^. Other than the occurrence of occasional cyclones between December and April, this tropical pelagic environment is likely to be more stable throughout the year than coastal waters, where seasonal changes in temperature are greater. Seasonal differences in the abundance of coastal bottlenose dolphins (*Tursiops* spp.) have been reported for some populations^48, 49^, but not others^50^.

There is a perception by the fishing industry and fisheries managers that dolphin abundance in the PTF is high, which may have arisen from observations of the high numbers of dolphins behind trawlers toward the end of each trawl (along with the assumption that these were ‘new’ dolphins each trawl) during fishing and the various bycatch surveys conducted in the PTF^26, 32^. Indeed, independent observers estimated group sizes at the time of winch-up of 25–50 dolphins^30^ and, in the current study; group sizes of 16–46 individuals were documented around one trawler. This study also showed that the mean group size of trawler-associated dolphins (28 individuals) was five or more times greater than that of dolphin groups observed from the air and not in the vicinity of trawlers (5 individuals). Furthermore, the aggregations of dolphins observed behind trawlers frequently include a proportion of the same individuals (see below). Moreover, Indo-Pacific bottlenose dolphins (*T. aduncus*), which may appear similar to those associating with the PTF, are often seen in north-western Australian coastal waters^43^. Allen *et al*.^29^, however, illustrated that the dolphins interacting with the PTF are a different species and genetically isolated from the coastal Indo-Pacific bottlenose dolphin populations and this study shows that the number of common bottlenose dolphins (*T. truncatus*) interacting with the PTF is relatively small. Determinations such as “Given the area of distribution and expected population size of these protected species, the impact of the trawl on the stocks of these protected species is probably minimal”; that up to 75 dolphin mortalities year^−1^ is an “acceptable” limit; and that mortalities from bycatch pose “negligible risk” to the dolphin population^32, 35, 51^ have been made in the absence of fundamental data on mortality rates and abundance, and appear to be overly optimistic.

The dedicated photo-identification effort, spanning some 60 trawls over two weeks, identified only 136 individual dolphins. More than a third of these dolphins (50 individuals) were photographed three to seven times, and estimates of the total number of dolphins associating with one of the three trawlers in the fleet varied from 183 to 226, depending on the type of model used. This represents just 8–10% of the total abundance of dolphins across the entire area of the PTF, as estimated by the concurrent aerial survey. If similar numbers were associating with the other two trawlers operating in the fishery at the time, the proportion is still only a maximum of 30%.Although these data are limited, the number of individuals resighted over separate ‘capture periods’ suggests that a proportion of the dolphin population show fidelity to trawler-associated foraging over days and weeks. This finding parallels that from Jaiteh *et al*.^30^, who found that individual dolphins were resighted on video footage collected inside a trawl net during different days and between separate fishing trips in the PTF over days and weeks.

During the dedicated survey, we photographically and genetically matched a number of individuals with those from opportunistic sampling in the PTF of up to 2.5 years earlier. Each individual match occurred within 140 km of the initial event, regardless of the time between the events; one individual moved 70 km in one day, and another was sampled just 15 km from where it was sampled 2.5 years earlier. It can be inferred from these data that at least some individuals also show fidelity to foraging behind trawlers over months to years. Resident communities of bottlenose dolphins (both *T. truncatus* and *T. aduncus*) are known to develop foraging traditions over years and between multiple generations^52, 53^, sometimes in association with trawl fisheries^54^. Many coastal bottlenose dolphin populations (again, both *Tursiops* spp.) consist largely of residents to a particular area^28^, although movements in the order of hundreds to over a thousand kilometres have been reported for some individuals (e.g., Greece^55^; United Kingdom^56^; southern California and north-western Mexico^57^). Far less is known of the residency and movements of pelagic *T. truncatus* populations. A few individuals have been documented moving considerable distances in short periods, i.e. thousands of kilometres^58^, but some offshore populations appear to include individuals with discrete home ranges and that display long-term site fidelity^59^. While based on a limited number of opportunistically collected samples, the results from the current study suggest movements by trawler-associated *T. truncatus* in north-western Australia are in the order of tens to hundreds of kilometres only, and a strong degree of fidelity to foraging around trawlers for at least a proportion of the community associating with the PTF.

In terms of the limitations of this study: Currently, we are not able to correct for availability bias in the estimate of dolphin abundance across the fishery; the capture-recapture estimate of trawler-associated dolphins was based on photo-identification of dolphins around only one of three trawlers operating in the fishery at the time; and the long-term photographic and genetic re-sampling was limited to <10 individuals. Thus, our measures of abundance and each measure of fidelity to trawler-associated foraging are likely to be under-estimates. Further research on these topics is warranted to reduce the uncertainty in the abundance estimates from the current study.

The abundance and density of bottlenose dolphins interacting with the PTF off north-western Australia appears to be lower than in comparable regions in the Gulf of Mexico, and similar in magnitude to some heavily degraded areas in the Mediterranean Sea. There are a number of plausible reasons for this, including being unable to account for availability bias resulting in an under-estimate of abundance, possible marked differences in productivity between regions, that historical and ongoing dolphin bycatch has impacted dolphin abundance, or a combination of these factors. Even if abundance was an order of magnitude greater than estimated in the current study, however, this dolphin population, or at least the community that interacts with the PTF, is subject to bycatch levels in trawl nets similar in magnitude to those in the western North Atlantic off the USA’s east coast, where the minimum population estimate of *T. truncatus* exceeds 55,000 offshore individuals^60^.

The combined elements of this study show that the number of dolphins interacting with the PTF is likely to be smaller than previously believed, and that at least a proportion of this community displays a high degree of fidelity to trawler-associated foraging over days, months and years. The fact that these dolphins are strongly motivated to interact with the trawlers and fishing gear increases the risk of entanglement above what might be expected from estimates based on random encounters between dolphins and trawlers. Independent research^25^ suggests dolphin capture rates of ≈50 dolphins year^−1^. Nevertheless, successive State and Commonwealth governments have determined “acceptable” dolphin bycatch limits and granted accreditation to the PTF in the absence of precise data on mortality rates and fundamental data on dolphin population size, or its ability to absorb such a rate of fishery-induced mortality. The results of the current research should be used to better-inform future management of the PTF and its impacts on populations of endangered, threatened and protected species.

In order to better understand the community/population of dolphins and its vulnerability to population-level impacts from bycatch in the PTF, we make the following recommendations:

- Further estimates of dolphin abundance are required in order to establish trends and rigourously assess the risks of ongoing bycatch. The use of unmanned aerial vehicles (UAVs) for this purpose may be more accurate, free of risk to humans and less costly than manned aerial surveys^61^. Furthermore, data on the surfacing intervals and dive times of common bottlenose dolphins in the PTF are required in order to correct for availability bias in future abundance estimation and reduce some of the sources of uncertainty in the estimates. With rapid advancements in the accessibility and ease of use of small UAVs, estimating availability could be achieved by operating from a trawler and/or a small research vessel.
- The re-commencement of an independent (human) observer program is required to estimate total bycatch objectively with greater precision than has been achieved with self-reporting^25^, as well as to validate the recent estimates based on electronic (video) monitoring^32^. This would provide the information necessary to estimate total fishery-induced mortality by including factors such as under-reported bycatch and the unobserved loss of dead or moribund dolphins during trawling.
- Samples should be gathered from common bottlenose dolphins in adjacent areas to assess levels of gene flow, or the degree of isolation of the fishery-impacted community^29^, and to better-define population boundaries and/or establish management or conservation units^62^.
- A prescriptive limit, established according to internationally accepted standards (e.g., Population Biological Removal^20, 63^), should be placed on the number of human-caused dolphin mortalities, beyond which management intervention should be triggered.
- This information should be used in combination with data on the biology of bottlenose dolphins (generation time, reproductive output) to conduct a Population Viability Analysis^63, 64^ before further assumptions are made by fisheries management agencies regarding the population’s conservation status^65^.

## Methods

### Pilbara Trawl Fishery

The PTF extends from longitude 116° E to 120° E and within the approximate boundaries of the 50 m depth contour to landward and the 100 m depth contour to seaward (Fig. 1). Four management areas (1, 2, 4 and 5) are open to trawling in the PTF, covering an area of ≈ 23,000 km^2^ (Fig. 1). One management area (3) is closed to trawling. Fishing trips last one to two weeks and occur year-round, with breaks in the Austral summer in the event of tropical cyclones. Between ≈ 7,300 and 10,300 h of trawling were conducted each year from 2010 to 2012^35^. The research activities described here had no influence on the operations of the fleet’s three trawlers.

### Aerial survey of the Pilbara Trawl Fishery to assess dolphin abundance

Eleven aerial survey flights were carried out between 11^th^ and 22^nd^ April, 2011, in a Cessna C337 (twin engine, overhead wing) aircraft. Four of the five PTF management areas (1, 2, 3 and 4) were surveyed, covering a total area of ≈18,250 km^2^ (Fig. 1). Operational difficulties prevented management area 5 from being surveyed. It is the furthest from the fishing ports and its extremities are subsequently subject to the least trawling activity, but no differences in bycatch rates were detected between management areas^25^. We therefore had no reason to expect marked differences in dolphin abundance between management areas. A series of 81 transects, oriented NW-SE and spaced 3.7 km (2 nm) apart (combined linear distance = 4,937.4 km), were surveyed by two port side observers (one fore and one aft), at an altitude of 152.4 m (500 feet) and a cruising speed of 185 km h^−1^ (100 knots), as per Dawson *et al*.^66^. Transects lines were approximately perpendicular to the coastline and depth contours, and parallel to the expected onshore/offshore density gradient for the species^67^. The speed of the aircraft was much faster than that of any animal movement, effectively providing a snapshot of abundance of animals at the surface in the area at the time of the survey^68^.

Observations were made from a double platform configuration, with the front and back observers visually and acoustically isolated from each other during the survey. The observers measured vertical angles from the plane to each sighted dolphin group using hand-held clinometers as the animals passed abeam of the aircraft. Based on known heights and vertical angles, perpendicular distances were calculated using trigonometry^69^. In addition to perpendicular distance, the following variables were recorded: (1) *Group size* (*S*) - observers provided three estimates (minimum, maximum, best); (2) *Fatigue* (*F*) - a measure of the time elapsed (in min) since the start of each flight; (3) *Time of day* (*T*) - a factor with two levels (morning = AM/afternoon = PM); (4) *Beaufort sea state* (*B*) - a factor with two levels (“low” for sea states ≤2, “high” if >2); (5) *Calves* (*CA*) - a binary factor coding for the presence (1)/absence (0) of one or more calves within the group; (6) *Cloud cover* (*CC*) - a factor with eight levels (one for each of 8 oktas); (7) *Glare intensity* (*G*) - a factor with four levels (0 = no glare, 1 = weak, 2 = moderate, 3 = high); (8) *Glare angle (GA)* - the angle of glare within the observers’ field of view (e.g., if the glare extends from 270° to 310°, then the GA takes a value of 310–270 = 40°).

All variables, and some of their interactions, were considered as covariates in fitting detection models. Surveys were undertaken in passing mode, although a circling protocol was employed when large (>15 animals) or trawler-associated groups were encountered, whereby the aircraft deviated from the transect line and circled the dolphins multiple times to confirm group size, composition and species identification^45^. Once these characteristics were confirmed, data collection along the transect line was resumed.

All analyses were carried out using the software package Distance 6.2 Release 1, available from http://www.ruwpa.st-and.ac.uk/distance/ ^70^. All unidentified and trawler-associated dolphins were excluded from these analyses, as these aggregations of dolphins could have unduly inflated the modelled abundance estimates. Mark-Recapture Distance Sampling (MRDS) models were fitted to the dual-observer sightings to estimate dolphin abundance. Further details on the modelling approach, the key assumptions of distance sampling and how they were addressed are available in the Supplementary Information (see also^71^). The multiple covariate distance sampling (MCDS) model estimates, based on all sightings (including those by the port observers and a single starboard observer) and the same truncation and filtering conditions as used in the MRDS analysis, were similar in magnitude, and are reported in Allen^71^.

### Photo-identification of trawler-associated dolphins to assess short-term fidelity

Bottlenose dolphins have natural markings on their dorsal fins, allowing the application of photo-identification methods for use in mark-recapture modelling to estimate abundance^72^. Two consecutive fishing trips on one trawler (of three) in the PTF were conducted between the 10^th^ and 25^th^ April, 2011, in fishery management areas 4 and 5 (Figs 1 and 2). Six of the seven photo-identification days coincided with aerial survey work. Twelve, 20-minute photo-identification surveys of individual, trawler-associated dolphins were undertaken (six surveys spread over four days during each fishing trip) from a 4.5 m inflatable boat deployed from the trawler ≈30 min before winch-up when conditions were favourable (sufficient light levels and Beaufort sea state ≤3). Mean trawl time in this fishery was ca. 2.7 h from 2003 to 2009^25^. Dolphins following the trawler were photographed randomly by making three or four passes of the group during each survey.

Two independent observers processed the photo-identification data from each sampling occasion by first quality grading each image and then cataloguing each distinctively marked individual. The best quality photograph for each individual captured on a sampling occasion was graded for quality in order to minimise misidentification and heterogeneity in capture probabilities^73^. The photographic quality grading protocol used is defined in Nicholson *et al*.^74^. Each individual in the catalogue was given a distinctiveness score, based on the amount of information contained on the leading and trailing edges of the dorsal fin. Only marks visible from both sides of the dorsal fin were used for identification, so that identifications made from photographs from either side of the dorsal fin could be included in the analyses.

Various capture-recapture models were run using program MARK^75^ to estimate the number of dolphins associated with the trawler, including: (1) simple closed models over all 12 sampling periods, given the surveys were conducted over only two weeks. In addition to the null model, models *M*_(t)_ and *M*_(th)_ were fitted to allow for any time (t) and individual variation (h) in capture probabilities; (2) a standard open model fitted using the Popan procedure in MARK^75, 76^ to allow for movement of animals in and out of the area (for further details, see the Supplementary Information and Allen^71^).

As the resulting abundance estimates were for the distinctively marked population around the trawler only, the proportion of marked individuals was estimated and the abundance estimates corrected to include the proportion of unmarked individuals in the population (see Supplementary Information).

### Opportunistic photographic and genetic sample matching to infer long-term fidelity

Individual dolphins photo-identified in April 2011 were compared with those photographed opportunistically on two previous trips aboard trawlers in October and November 2008, ranging in duration from seven to ten days. Biopsy samples were also collected opportunistically on four trips between October 2008 and April 2011 and were used to assess fidelity to foraging around trawlers over periods of months to years. Small tissue samples were collected, stored and analysed as described in Allen *et al*.^29^. The software microsatellite toolkit^77^ was used to determine identical genotypes among all sampled individuals. The locations of individuals that were re-sampled at least one day after their initial sampling were plotted within the management areas, noting the time and distance between events.

### Data availability statement

The datasets generated during and/or analysed during the current study are available from the corresponding author on reasonable request.

### Approvals

The Murdoch University Animal Ethics Committee approved all experimental protocols, and the research was carried out under permits for the scientific use of animals from both State (Department of Parks and Wildlife) and Commonwealth (Department of Environment) wildlife management agencies.

### Accordance

All research was carried out in accordance with the relevant guidelines and regulations.

## Electronic supplementary material

Supplementary Information

## References

[1] Read AJ, Drinker P, Northridge S (2006). Bycatch of marine mammals in U.S. and global fisheries. Conserv. Biol..

[2] Halpern BS, Selkoe KA, Micheli F, Kappel CV (2007). Evaluating and ranking the vulnerability of global marine ecosystems to anthropogenic threats. Conserv. Biol..

[3] FAO. *The State of World Fisheries and Aquaculture 2014* (Food and Agriculture Organization of the United Nations, Rome, 2014).

[4] Read AJ (2008). The Looming Crisis: Interactions between marine mammals and fisheries. J. Mammal..

[5] Lewison RL (2014). Global patterns of marine mammal, seabird, and sea turtle bycatch reveal taxa-specific and cumulative megafauna hotspots. Proc. Natl. Acad. Sci. USA..

[6] Turvey ST (2007). First human-caused extinction of a cetacean species?. Biol. Lett..

[7] Piroddi C, Bearzi G, Gonzalvo J, Christensen V (2011). From common to rare: The case of the Mediterranean common dolphin. Biol. Conserv..

[8] Mei Z (2012). Accelerating population decline of Yangtze finless porpoise (*Neophocaena asiaeorientalis asiaeorientalis*). Biol. Conserv..

[9] Robertson BC, Chilvers BL (2011). The population decline of the New Zealand sea lion *Phocarctos hookeri*: a review of possible causes. Mammal Rev.

[10] Hamer D (2013). The endangered Australian sea lion extensively overlaps with and regularly becomes by-catch in demersal shark gill-nets in South Australian shelf waters. Biol. Conserv..

[11] Worm B (2013). Global catches, exploitation rates, and rebuilding options for sharks. Mar. Policy.

[12] Jaramillo-Legorreta, A. *et al*. Passive acoustic monitoring of the decline of Mexico’s critically endangered vaquita. *Conserv. Biol*., doi:10.1111/cobi.12789 (2016). 10.1111/cobi.12789 27338145

[13] Jackson JBC (2001). Historical overfishing and the recent collapse of coastal ecosystems. Science.

[14] Pauly D (2002). Towards sustainability in world fisheries. Nature.

[15] Worm B (2006). Impacts of biodiversity loss on ocean ecosystem services. Science.

[16] Estes JA (2011). Trophic downgrading of Planet Earth. Science.

[17] Marsh, H. *et al*. Strategies for conserving marine mammals in *Marine Mammals: Fisheries, tourism and management issues* (eds Gales, N., Hindell, M. & Kirkwood, R.) 1–19 (CSIRO Publishing, 2003).

[18] Lewison R, Crowder L, Read A, Freeman S (2004). Understanding impacts of fisheries bycatch on marine megafauna. Trends Ecol. Evol..

[19] Cox TM (2007). Comparing effectiveness of experimental and implemented bycatch reduction measures: the ideal and the real. Conserv. Biol..

[20] Wade PR (1998). Calculating limits to the allowable human-caused mortality of cetaceans and pinnipeds. Mar. Mammal Sci..

[21] Roman J (2013). The Marine Mammal Protection Act at 40: status, recovery, and future of U.S. marine mammals. Ann. N.Y. Acad. Sci..

[22] Woinarski, J. C. Z., Burbidge, A. A. & Harrison, P. L. *The Action Plan for Australian Mammals 2012* (CSIRO Publishing, 2014).

[23] Harwood, M. B. & Hembree, D. Incidental catch of small cetaceans in the offshore gillnet fishery in Northern Australian waters: 1981-1985 in *Report 37*. 363–367 (International Whaling Commission, 1987).

[24] Hamer DJ, Ward TM, McGarvey R (2008). Measurement, management and mitigation of operational interactions between the South Australian Sardine Fishery and short-beaked common dolphins (*Delphinus delphis*). Biol. Conserv..

[25] Allen SJ (2014). Patterns of dolphin bycatch in a north-western Australian trawl fishery. PloS One.

[26] Stephenson, P. C. & Chidlow, J. *Bycatch in the Pilbara Trawl Fishery*. Final report to the Natural Heritage Trust (Western Australian Department of Fisheries, 2003).

[27] Taylor BL, Wade PR, De Master DP, Barlow J (2000). Incorporating uncertainty into management models for marine mammals. Conserv. Biol..

[28] Connor, R., Wells, R., Mann, J. & Read, A. The bottlenose dolphin: social relationships in a fission-fusion society in *Cetacean societies:* Field *studies of whales and dolphins* (eds Mann, J. Connor, R., Tyack, P. & Whitehead, H.) 91–126 (University of Chicago Press, 2000).

[29] Allen SJ (2016). Genetic isolation between coastal and fishery-impacted, offshore bottlenose dolphin (*Tursiops* spp.) populations. Mol. Ecol..

[30] Jaiteh VF, Allen SJ, Meeuwig JJ, Loneragan NR (2013). Subsurface behavior of bottlenose dolphins (*Tursiops truncatus*) interacting with fish trawl nets in northwestern Australia: Implications for bycatch mitigation. Mar. Mamm. Sci.

[31] Jaiteh VF, Allen SJ, Meeuwig JJ, Loneragan NR (2014). Combining in-trawl video with observer coverage improves understanding of protected and vulnerable species by-catch in trawl fisheries. Mar. Freshw. Res..

[32] Wakefield, C. B. *et al*. Independent observations of catches and subsurface mitigation efficiencies of modified trawl nets for endangered, threatened and protected megafauna bycatch in the Pilbara Fish Trawl Fishery. Fisheries Research report No. 244 (Western Australian Department of Fisheries, 2014).

[33] Ward, T., Ivey, A. & Burch, P. *Effectiveness of an industry Code of Practice in mitigating the operational interactions of the South Australian Sardine Fishery with the short-beaked common dolphin (Delphinus delphis)* (South Australian Research and Development Institute, 2012).

[34] Moore JE (2010). An interview-based approach to assess marine mammal and sea turtle captures in artisanal fisheries. Biol. Conserv..

[35] Fletcher, W. & Santoro, K. *Status reports of the fisheries and aquatic resources of Western Australia 2012/13* (Western Australian Department of Fisheries, 2013).

[36] Waring, G. *et al*. 1999. *US Atlantic and Gulf of Mexico marine mammal stock assessments - 1999*. NOAA Technical Memorandum (National Oceanic and Atmospheric Administration, 1999).

[37] Miller LJ, Mackey AD, Solangi M, Kuczaj SA (2013). Population abundance and habitat utilization of bottlenose dolphins in the Mississippi Sound. Aquat. Conserv.: Mar. Freshw. Ecosystems.

[38] Forcada J, Gazo M, Aguilar A, Gonzalvo J, Fernández-Contreras M (2004). Bottlenose dolphin abundance in the NW Mediterranean: addressing heterogeneity in distribution. Mar. Ecol. Prog. Ser..

[39] Cañadas A, Hammond PS (2006). Model-based abundance estimates for bottlenose dolphins off southern Spain: implications for conservation and management. J. Cetacean Res. Manage..

[40] Bearzi G, Holcer D, Notarbartolo di Sciara G (2004). The role of historical dolphin takes and habitat degradation in shaping the present status of northern Adriatic cetaceans. Aquat. Conserv.: Mar. Freshw. Ecosystems.

[41] Bearzi G, Politi E, Agazzi S, Azzellino A (2006). Prey depletion caused by overfishing and the decline of marine megafauna in eastern Ionian Sea coastal waters (central Mediterranean). Biol. Conserv..

[42] Lauriano G, Pierantonio N, Donovan G, Panigada S (2014). Abundance and distribution of *Tursiops truncatus* in the Western Mediterranean Sea: an assessment towards the Marine Strategy Framework Directive requirements. Mar. Environmental Res..

[43] Allen SJ, Cagnazzi DD, Hodgson AJ, Loneragan NR, Bejder L (2012). Tropical inshore dolphins of north-western Australia: Unknown populations in a rapidly changing region. Pacific Conserv. Biol..

[44] Thomson JA, Cooper AB, Burkholder DA, Heithaus M, Dill LM (2013). Correcting for heterogeneous availability bias in surveys of long-diving marine turtles. Biol. Conserv..

[45] Slooten E, Dawson S, Rayment W (2004). Aerial surveys for coastal dolphins: abundance of Hector’s dolphins on the South Island West Coast, New Zealand. Mar. Mammal Sci..

[46] Slooten E, Dawson S, Rayment W, Childerhouse S (2006). A new abundance estimate for Maui’s dolphin: What does it mean for managing this critically endangered species?. Biol. Conserv..

[47] Pollock KH, Marsh HD, Lawler IR, Alldredge MW (2006). Estimating animal abundance in heterogeneous environments: An application to aerial surveys for dugongs. J. Wildlife Manage..

[48] Balmer BC (2008). Seasonal abundance and distribution patterns of common bottlenose dolphins (*Tursiops truncatus*) near St. Joseph Bay, Florida, U.S.A. J. Cetacean Res. Manage..

[49] Sprogis, K. *et al*. Sex-specific patterns of abundance, temporary emigration and survival of Indo-Pacific bottlenose dolphins (*Tursiops aduncus*) in coastal and estuarine waters. *Fr. Mar. Sci*. **3**, 12, 10.3389/fmars.2016.00012.

[50] Williams JA, Dawson SM, Slooten E (1993). The abundance and distribution of bottlenosed dolphins (*Tursiops truncatus*) in Doubtful Sound, New Zealand. Canadian J. Zool..

[51] Department of Fisheries. *State of the Fisheries Report 2003/2004* (Western Australian Department of Fisheries, 2004).

[52] Daura-Jorge FG, Cantor M, Ingram SN, Lusseau D, Simões-Lopes PC (2012). The structure of a bottlenose dolphin society is coupled to a unique foraging cooperation with artisanal fishermen. Biol. Lett..

[53] Krützen M (2014). Cultural transmission of tool use by Indo-Pacific bottlenose dolphins (*Tursiops* sp.) provides access to a novel foraging niche. Proc. R. Soc. London B.

[54] Chilvers BL, Corkeron PJ (2001). Trawling and bottlenose dolphins’ social structure. Proc. R. Soc. London B.

[55] Bearzi G, Bonizzoni S, Gonzalvo J (2010). Mid-distance movements of common bottlenose dolphins in the coastal waters of Greece. J. Ethology.

[56] Robinson K (2012). Discrete or not so discrete: Long distance movements by coastal bottlenose dolphins in UK and Irish waters. J. Cetacean Res. Manage..

[57] Hwang A, Defran RH, Bearzi M, Maldini D (2014). Coastal range and movements of common bottlenose dolphins (*Tursiops truncatus*) off California and Baja California, Mexico. Bulletin Southern California Acad. Sci..

[58] Wells RS (1999). Long distance offshore movements of bottlenose dolphins. Mar. Mammal Sci..

[59] Rossbach KA, Herzing DL (1999). Inshore and offshore bottlenose dolphin (*Tursiops truncatus*) communities distinguished by association patterns near Grand Bahama Island, Bahamas. Canadian J. Zool..

[60] Waring, G. T. *et al*. *US Atlantic and Gulf of Mexico Marine Mammal Stock Assessments - 2013*. NOAA Technical Memorandum (National Oceanic and Atmospheric Administration, 2014).

[61] Hodgson A, Kelly N, Peel D (2013). Unmanned aerial vehicles (UAVs) for surveying marine fauna: a dugong case study. PloS One.

[62] Palsbøll PJ, Bérubé M, Allendorf FW (2007). Identification of management units using population genetic data. Trends Ecol. Evol..

[63] Thompson PM, Wilson B, Grellier K, Hammond PS (2000). Combining power analysis and population viability analysis to compare traditional and precautionary approaches to conservation of coastal cetaceans. Conserv. Biol..

[64] Shaffer ML (1990). Population viability analysis. Conserv. Biol..

[65] Wakefield, C. B. *et al*. Performance of bycatch reduction devices varies for chondrichthyan, reptile, and cetacean mitigation in demersal fish trawls: assimilating subsurface interactions and unaccounted mortality. *ICES J. Mar. Sci*. 10.1093/icesjms/fsw143 (2016).

[66] Dawson S, Wade P, Slooten E, Barlow J (2008). Design and field methods for sighting surveys of cetaceans in coastal and riverine habitats. Mammal Rev..

[67] Buckland, S. *et al*. Introduction to distance sampling: Estimating abundance of biological populations (Oxford University Press, 2001).

[68] Buckland, S. *et al*. Advanced distance sampling: Estimating abundance of biological populations (Oxford University Press, 2004).

[69] Lerczak G, Hobbs R (1998). Calculating sighting distances from angular readings during shipboard, aerial, and shore-based marine mammal surveys. Mar. Mammal Sci..

[70] Thomas L (2010). Distance software: design and analysis of distance sampling surveys for estimating population size. J. Appl. Ecol..

[71] Allen, S. J. Fishery-impacted bottlenose dolphins of north-western Australia: Bycatch patterns, genetic status and abundance. Doctoral thesis (Murdoch University, 2015).

[72] Würsig B, Würsig M (1977). The photographic determination of group size, composition and stability of coastal porpoises (*Tursiops truncatus*). Science.

[73] Friday N, Smith T, Stevick P, Allen J (2000). Measurement of photographic quality and individual distinctiveness for the photographic identification of humpback whales. Megaptera novaeangliae. Mar. Mammal Sci..

[74] Nicholson K, Bejder L, Allen SJ, Krützen M, Pollock KH (2012). Abundance, survival and temporary emigration of bottlenose dolphins (*Tursiops* sp.) off Useless Loop in the western gulf of Shark Bay, Western Australia. Mar. Freshw. Res..

[75] White G, Burnham K (1999). Program MARK: survival estimation from populations of marked animals. Bird Study.

[76] Williams, B., Nichols, J. & Conroy, M. *Analysis and management of animal populations* (Academic Press, 2002).

[77] Park, S. Trypanotolerance in West African cattle and the population genetic effects of selection. Doctoral thesis (University of Dublin, 2001).

