# Supplementary material for: Preliminary estimates of the abundance and fidelity of dolphins associating with a demersal trawl fishery
Source: Sci Rep. 2017 Jul 10;7:4995. doi: 10.1038/s41598-017-05189-0 (PMC5503993; doi:10.1038/s41598-017-05189-0)
Supplement: Supplementary file 1 — Supplementary Information [file 41598_2017_5189_MOESM1_ESM.doc]

**Preliminary estimates of the abundance and fidelity of dolphins associating with a demersal trawl fishery - Supplementary Information**

Simon J. Allen, Kenneth H. Pollock, Phil J. Bouchet, Halina T. Kobryn, Deirdre B. McElligott, Krista E. Nicholson, Joshua N. Smith, Neil R. Loneragan

***Aerial survey of the Pilbara Trawl Fishery to assess dolphin abundance***

*Data truncation, filtering and analyses*

All analyses were carried out using the open-source software package Distance 6.2 Release 1, available from http://www.ruwpa.st-and.ac.uk/distance/. As often occurs in aerial surveys of marine animals, the flat windows of the Cessna limited visibility in the vicinity of the flight path. To remedy this, sightings were left-truncated at 100 m (threshold chosen based on a visual inspection of the raw data). Although automatic left-truncation is implemented within Distance 6.2, the software assumes that detection is certain on the trackline and, therefore, extrapolates the detection function back to distance 0. Left-truncation was performed manually (in Microsoft Excel) by discarding sightings located within the first 100 m and subtracting “100” from the measured distances of all remaining data points (as per^1^). Data were subsequently binned into 75 m intervals and right-truncated at 450 m. All unidentified and trawler-associated clusters of dolphins were excluded from this analysis so as not to unduly inflate the abundance estimates. Mark-recapture distance sampling (MRDS) models were fitted to the dual-observer sightings on the port side to estimate the abundance of available dolphins whilst accounting for any departures from the key assumption (see below) that g(0) (detection probability on the trackline) is equal to 1.

A total of 69 plausible candidate models were tested under both assumptions of point (with a half-normal key function) and full independence^2,3^. A logistic form was used to derive conditional detection probability functions p_1|2_(y, *z*) (probability that observer 1 detects an animal, given that it is seen by observer 2) and p_2|1_(y, *z*) (probability that observer 2 detects an animal, given that it is seen by observer 1)^4^. The overall probability *p.* that at least one observer detects an animal was then calculated as:

p.(y, *z*) = p_1_(y, *z*) + p_2_(y, *z*) [1 – p_1|2_(y, *z*)]

(where z is a vector of explanatory terms and y represents distance)^5^.

Models included multiple combinations of covariate main effects and their first-order interactions and were selected based on the Akaike's information criterion (AIC). Since the Distance program for MRDS estimation does not currently enable model averaging, the model with minimum AIC score was retained as the final best model. These models were used to provide the best possible abundance estimate under the constraints of the available data (both in terms of sample sizes and available covariates recorded^5^).

All Full Independence (FI) models exhibited lower AIC scores than Point Independence (PI) models and were, thus, preferred (Supplementary Table S1). While PI models have generally been favoured as more robust than their FI equivalent in the literature, FI models can prove equally as appropriate (or more so) in a number of situations^4-6^. Smaller sample sizes favour the simpler models with the stronger assumptions. In these conditions, fitting a PI model can lower bias, but it reduces precision and requires the estimation of more parameters.

***Supplementary Table S1: Model details and selection results for the mark-recapture distance sampling, based on bottlenose dolphin dual observer data. The best FI model (selected based on AIC scores) is shown in bold. Covariate terms are as follows: Dist = distance, B = Beaufort sea state, CA = calf presence, CC = cloud cover, F = fatigue, G = glare intensity, GA = glare angle, S = group size, T = Time of day. Colons “:” code for variable interactions. Derived parameters include animal density (D), abundance (N) and associated 95% CI (N_low_; N_high_).***

|  | **Model** | **Point Independence** | | |  |  |  |  | **Full Independence** | | |  |  |  |
| --- | --- | --- | --- | --- | --- | --- | --- | --- | --- | --- | --- | --- | --- | --- |
|  |  | **g. (y, z)** | **N** | **N_low_** | **N_high_** | **D** | **AIC** | **ΔAIC** | **ΔAIC** | **N** | **N_low_** | **N_high_** | **D** | **AIC** |
| 1 | Dist | 1 | 1,823 | 1,090 | 3,049 | 0.09988 | 192.6 | 82.4 | 6.4 | 1,321 | 757 | 2,306 | 0.07237 | 116.6 |
| 2 | Dist | CC | 2,104 | 1,170 | 3,785 | 0.11528 | 192.9 | 82.7 | - | - | - | - | - | - |
| 3 | Dist | GA | 1,897 | 1,086 | 3,313 | 0.10395 | 194.8 | 84.6 | - | - | - | - | - | - |
| 4 | Dist | S | 1,646 | 1,000 | 2,709 | 0.09019 | 192.5 | 82.3 | - | - | - | - | - | - |
| 5 | Dist | T | 1,817 | 1,085 | 3,045 | 0.09957 | 194.5 | 84.3 | - | - | - | - | - | - |
| 6 | Dist | S+G | 1,686 | 908 | 3,131 | 0.09236 | 196.9 | 86.7 | - | - | - | - | - | - |
| 7 | Dist | S+T | 1,643 | 992 | 2,719 | 0.09000 | 194.4 | 84.3 | - | - | - | - | - | - |
| 8 | Dist | S+B | 1,691 | 1,017 | 2,814 | 0.09267 | 194.2 | 84.1 | - | - | - | - | - | - |
| 9 | Dist+B | S | 1,648 | 1,001 | 2,714 | 0.09029 | 194.3 | 84.1 | 8.6 | 1,320 | 754 | 2,311 | 0.07231 | 118.8 |
| 10 | Dist+CA | S | 1,655 | 1,004 | 2,728 | 0.09069 | 192.0 | 81.8 | 6.7 | 1,331 | 759 | 2,334 | 0.07291 | 116.9 |
| 11 | Dist+CC | S | 1,647 | 1,001 | 2,709 | 0.09021 | 193.5 | 83.3 | 8.8 | 1,581 | 742 | 3,367 | 0.08662 | 119.0 |
| 12 | Dist+F | S | 1,650 | 1,002 | 2,718 | 0.09041 | 193.4 | 83.2 | 6.1 | 1,370 | 768 | 2,443 | 0.07506 | 116.3 |
| 13 | Dist+G | S | 1,677 | 1,011 | 2,781 | 0.09188 | 194.0 | 83.8 | 4.2 | 1,526 | 797 | 2,922 | 0.08362 | 114.4 |
| 14 | Dist+GA | S | 1,652 | 1,001 | 2,726 | 0.09049 | 193.4 | 83.2 | 6.5 | 1,357 | 755 | 2,439 | 0.07436 | 116.7 |
| 15 | Dist+S | S | 1,643 | 999 | 2,703 | 0.09001 | 194.4 | 84.2 | 7.7 | 1,314 | 756 | 2,284 | 0.07199 | 117.9 |
| 16 | Dist+T | S | 1,647 | 1,002 | 2,706 | 0.09023 | 193.2 | 83.0 | 4.4 | 1,369 | 780 | 2,401 | 0.07500 | 114.6 |
| 17 | Dist+T+B | S | 1,648 | 1,003 | 2,709 | 0.09028 | 195.2 | 85.0 | 6.7 | 1,366 | 777 | 2,401 | 0.07484 | 116.9 |
| 18 | Dist+T+CA | S | 1,654 | 1,006 | 2,721 | 0.09064 | 193.2 | 83.0 | 5.7 | 1,366 | 777 | 2,401 | 0.07484 | 115.9 |
| 19 | Dist+T+F | S | 1,653 | 1,006 | 2,716 | 0.09055 | 193.2 | 83.0 | 1.2 | 1,484 | 814 | 2,705 | 0.08129 | 111.4 |
| 20 | Dist+T+G | S | 1,680 | 1,017 | 2,776 | 0.09205 | 195.0 | 84.8 | 2.9 | 1,578 | 818 | 3,046 | 0.08647 | 113.1 |
| 21 | Dist+T+GA | S | 1,651 | 1,003 | 2,716 | 0.09043 | 193.5 | 83.3 | 2.7 | 1,430 | 790 | 2,587 | 0.07835 | 112.9 |
| 22 | Dist+T+S | S | 1,646 | 1,002 | 2,705 | 0.09018 | 195.2 | 85.0 | 6.2 | 1,367 | 779 | 2,399 | 0.07487 | 116.4 |
| 23 | Dist+B+G | S | 1,684 | 1,013 | 2,799 | 0.09228 | 195.8 | 85.6 | 6.6 | 1,532 | 791 | 2,967 | 0.08393 | 116.8 |
| 24 | Dist+S+G | S | 1,669 | 1,010 | 2,759 | 0.09143 | 195.9 | 85.7 | 5.3 | 1,491 | 792 | 2,809 | 0.08170 | 115.5 |
| 25 | Dist+G+F | S | 1,679 | 1,011 | 2,788 | 0.09197 | 195.8 | 85.6 | 5.4 | 1,554 | 799 | 3,022 | 0.08514 | 115.6 |
| 26 | Dist+S+B | S | 1,645 | 999 | 2,707 | 0.09010 | 196.2 | 86.0 | 9.8 | 1,313 | 753 | 2,288 | 0.07191 | 120.0 |
| 27 | Dist+S+F | S | 1,646 | 1,000 | 2,707 | 0.09016 | 195.3 | 85.1 | 7.1 | 1,356 | 767 | 2,398 | 0.07429 | 117.3 |
| 28 | Dist+S+GA | S | 1,649 | 1,000 | 2,721 | 0.09036 | 195.4 | 85.2 | 8.1 | 1,349 | 751 | 2,424 | 0.07390 | 118.3 |
| 29 | Dist+T+F+S | S | 1,650 | 1,004 | 2,711 | 0.09040 | 195.2 | 85.0 | 2.7 | 1,473 | 810 | 2,679 | 0.08072 | 112.9 |
| **30** | **Dist+T+F+GA** | S | 1,656 | 1,006 | 2,728 | 0.09075 | 193.7 | 83.5 | **0.0** | **1,551** | **822** | **2,929** | **0.08499** | **110.2** |
| 31 | Dist T+F+G | S | 1,679 | 1,016 | 2,776 | 0.09201 | 196.3 | 86.1 | 2.8 | 1,633 | 833 | 3,203 | 0.08948 | 113.0 |
| 32 | Dist+T+F+CA | S | 1,659 | 1,009 | 2,729 | 0.09089 | 193.6 | 83.4 | 3.3 | 1,463 | 804 | 2,660 | 0.08013 | 113.5 |
| 33 | Dist T+F+GA+F:T | S | 1,657 | 1,005 | 2,733 | 0.09079 | 195.7 | 85.5 | 2.1 | 1,551 | 821 | 2,930 | 0.08497 | 112.3 |
| 34 | Dist+T+F+GA+F:GA | S | 1,699 | 981 | 2,945 | 0.09311 | 194.7 | 84.5 | 3.2 | 1,609 | 680 | 3,805 | 0.08814 | 113.4 |
| 35 | Dist+T+F+GA+F:GA+F:T | S | 1,709 | 981 | 2,978 | 0.09363 | 196.6 | 86.4 | 6.3 | 1,604 | 676 | 3,803 | 0.08786 | 116.5 |
| 36 | Dist+T+F+S+S:F | S | 1,762 | 972 | 3,193 | 0.09654 | 193.6 | 83.4 | 3.0 | 1,855 | 646 | 5,325 | 0.10163 | 113.2 |
| 37 | Dist+T+F+B+F:B | S | 1,651 | 1,004 | 2,712 | 0.09043 | 196.7 | 86.5 | 3.2 | 1,520 | 827 | 2,793 | 0.08325 | 113.4 |
| 38 | Dist T+F+GA+S+S:F | S | 1,768 | 964 | 3,243 | 0.09687 | 194.3 | 84.1 | 2.3 | 1,989 | 640 | 6,182 | 0.10899 | 112.5 |

The average trackline detection probability *p(0)* for each observer was 0.834 and 0.962 for both observers combined (Supplementary Fig. S1). The estimated *p(0)* is only marginally lower than 1 and a standard conventional distance sampling analysis (with a constrained half-normal key function) yielded a comparable abundance estimate of 1,596 animals, albeit with a small gain in precision (95% CI = 932-2,736).


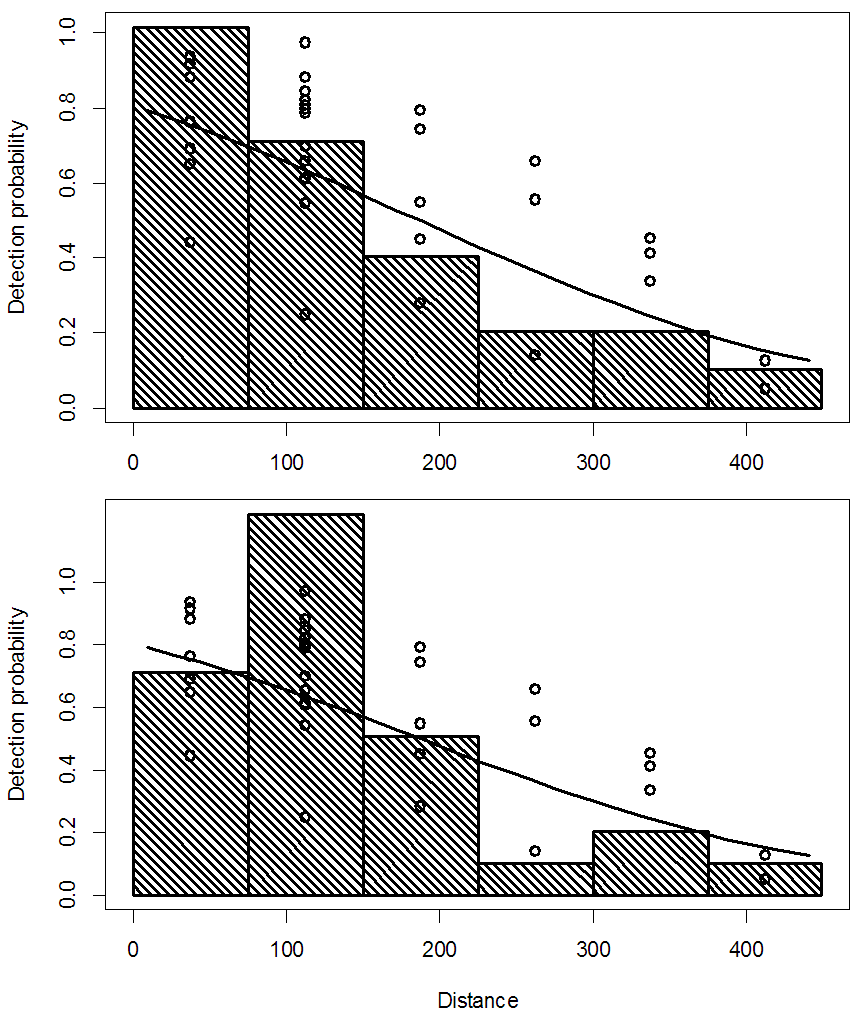


**Supplementary Figure S1.** Fitted detection probability models for observer 2 (top) and observer 1 (bottom).

*Addressing different cluster sizes in the field and program distance*

Dolphins occur in clusters that can vary considerably in size and, therefore, detectability. Clusters, defined here as one or more bottlenose dolphins, were accurately recorded by using experienced observers and employing a circle-back protocol for group sizes of ≥ 15 individuals. This also allowed for species identifications and cluster sizes to be confirmed. Program Distance estimates mean cluster size using the regression method, in which log cluster size is regressed on estimated probability of detection^7^. This removes any size bias effect, i.e. larger clusters are easier to detect than small clusters at greater distances, so the simple mean of observed cluster sizes is a positively biased estimate of population mean cluster size. This method also corrects for any bias that might arise if cluster size is underestimated at greater distances, such that the mean observed cluster size would be a negatively biased estimate of population mean cluster size^8^.

*Assumptions of distance sampling methods*

There are three key assumptions in distance sampling: (1) Objects on the line are detected with certainty; (2) Objects do not move; and (3) Measurements are exact^7,8^. These assumptions are addressed above and by using trained, experienced observers and clinometres^1,9,10^.

***Photo-identification of trawler-associated dolphins to assess short-term fidelity***

*Modelling approach*

Various capture-recapture models to estimate the abundance of trawler-associated dolphins were run using program MARK^11^, starting with:

1. The simplest closed models over all 12 sampling periods, given the surveys were conducted over only two weeks. Models *M*_(t)_ and *M*_(th)_ were fitted to allow for any time (t) and individual variation (h) in capture probabilities.
2. While the study was short, a lack of population closure was expected due to the spatial nature of the sampling over a sizeable area. A standard open model was therefore fitted using the Popan procedure in MARK^11,12^.
3. One drawback of the open models is that they do not allow for temporary emigration, which may have occurred here. Hence, Robust Design models were also fitted, which allow for temporary emigration. Using the full data set, the 12 sampling events were grouped into five primary periods (1 = 1, 2, 3; 2 = 4, 5; 3 = 6, 7, 8; 4 = 9, 10; and 5 = 11, 12) by spatial proximity. Furthermore, a reduced data set of 10 survey events was grouped into four primary periods (1 = 1, 2, 3; 2 = 4, 5; 3 = 7, 8; and 4 = 9, 10) by temporal proximity (i.e., days within which multiple sampling events occurred).

*Proportion of distinctly marked trawler-associated dolphins*

The proportion of distinctly marked individuals (
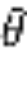

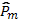
) in the population was estimated as the proportion of photographs showing a distinctly marked (*D1* and *D2*) individual from a random sample of 300 good quality (QS < 12) photographs. With this estimation, we assumed that photographs were taken randomly during each sampling occasion. Some bias may have been introduced, however, by a tendency to focus on more distinctly marked individuals. Calves were excluded from this estimation.

*Total abundance of trawler-associated dolphins*

The population size estimates from the capture-recapture models relate to the distinctly marked population. To estimate the total population size at a particular time, these estimates were adjusted to take into account the proportion of individuals in the population that are unmarked:


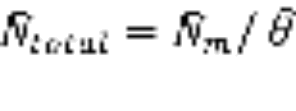
.

Here,
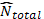
 is the estimated total population size,
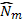
 the estimated distinctly marked population size and
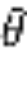
 the estimated proportion of distinctly marked individuals in the population. The approximate variance for the estimated total population size was derived using the following formula for the standard error of a ratio:


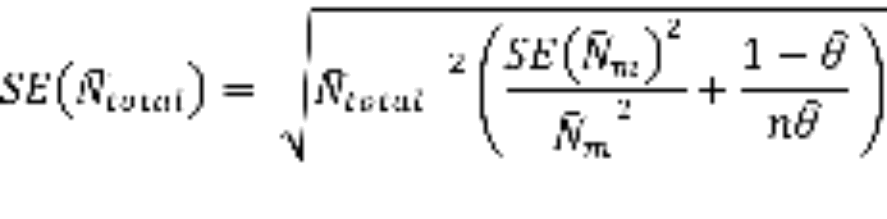


using the delta method^12^. Log-normal 95% confidence intervals were calculated with a lower limit of
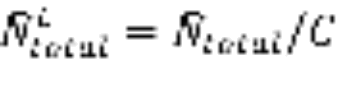
 and upper limit of
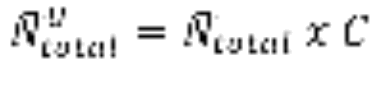
, where:


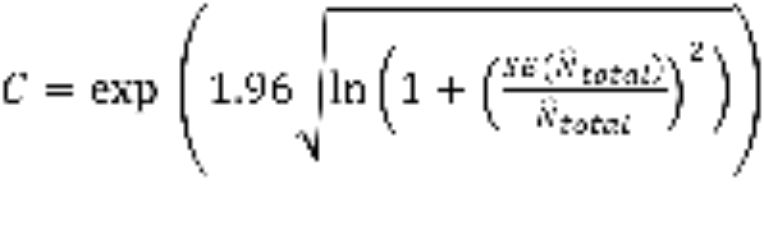
13

*Addressing the assumptions of the Robust Design method*

The assumptions of this application of the Robust Design method were:

(1) All individuals had equal probability of being captured within a secondary sampling occasion,

(2) Capture and recapture probabilities were equal (no trap response),

(3) Marks were unique, permanent and identified correctly,

(4) The sampling interval for a secondary sample was instantaneous,

(5) The population was closed within primary periods,

(6) All individuals have equal probability of survival, and

(7) Each individual’s probability of capture was independent of all others^12,14^.

Regardless of the sampling methods used, the assumption of all individuals having equal probability of capture is not often met for cetacean populations^15^. In this study, however, the “population” being “captured” was an aggregation of dolphins foraging behind a trawler. All individuals attracted to the trawler were likely to have had an equal probability of being photographed (“captured”). The assumption of the first capture and then recapture probabilities being equal was considered valid as photo-identification does not require physical capture or handling of animals^16^. Capture probabilities can be heterogeneous because of an individual’s age, sex or social status, and some individuals may be more distinctly marked than others. Such sources of heterogeneity were minimized by; conducting three to four repeated passes of the aggregations of dolphins behind the trawler in each 20-min sampling occasion; by including only captures from excellent- and good-quality photographs; and by including only sufficiently marked individuals in the analyses^17^. The secondary sampling intervals were considered instantaneous and population closure within primary periods was assumed, given their brief duration and spatial and temporal proximity relative to bottlenose dolphin life history and ranging characteristics. Finally, pelagic bottlenose dolphin populations are not known to be socially structured to the extent of coastal populations^18^ and our protocol was such that capturing (i.e., photographing) a particular individual would be unlikely to effect the chance of capturing its close associates.

***References***

1. Borchers, D. L, Laake, J. L., Southwell, C. & Paxton, C. G. M. Accommodating unmodeled heterogeneity in double-observer distance sampling surveys. *Biometrics* **62**, 372-378 (2006).
2. Laake, J., Dawson, M. & Hone, J. Visibility bias in aerial survey: Mark-recapture, line-transect or both? *Wildlife Res.* **35**, 299-309 (2008).
3. Fewster, R. & Pople, A. A comparison of mark-recapture distance-sampling methods applied to aerial surveys of eastern grey kangaroos. *Wildlife Res.* **35**, 320-330 (2008).
4. Buckland, S., Laake, J. & Borchers, D. Double-observer line transect methods: Levels of independence. *Biometrics* **66**, 169-177 (2009).
5. Buckland, S., *et al*. *Advanced distance sampling: Estimating abundance of biological populations* (Oxford University Press, 2004).
6. Collier, B. A., *et al.* Modeling spatially explicit densities of endangered avian species in a heterogeneous landscape. *The Auk* **130**, 666-676 (2013).
7. Buckland, S., *et al.* *Introduction to distance sampling: Estimating abundance of biological populations* (Oxford University Press, 2001).
8. Thomas, L., *et al.* Distance software: design and analysis of distance sampling surveys for estimating population size. *J. Appl. Ecol.* **47**, 5-14 (2010).
9. Alldredge, M. W., Simons, T. R. & Pollock, K. H. A field evaluation of distance measurement error in auditory avian point count surveys. *J. Wildlife Manage.* **71**, 2759–2766 (2007).
10. Salgado Kent, C., Jenner, K., Jenner, M.-N., Bouchet, P. & Rexstad, E. Southern Hemisphere breeding stock "D" humpback whale population estimates from North West Cape, Western Australia. *J. Cetacean Res. Manage.* **12**, 29-38 (2012).
11. White, G. C. & Burnham, K. P. Program MARK: survival estimation from populations of marked animals. *Bird Study* **46**, 120–139 (1999).
12. Williams, B. K., Nichols, J. D. & Conroy, M. J. *Analysis and Management of Animal Populations* (Academic Press, San Diego, 2002).
13. Burnham, K. P., Anderson, D. R., White, G. C., Brownie, C. & Pollock, K. H. Design and analysis methods for fish survival experiments based on release-recapture. *American Fish. Soc. Monogr. 5* (Bethesda, 1987).
14. Pollock, K. H., Nichols, J. D, Brownie, C. & Hines, J. E. Statistical inference for capture–recapture experiments. *Wildlife Monogr.* **107**, 3–97 (1990).
15. Hammond, P. S. Estimating the size of naturally marked whale populations using capture-recapture techniques. *Rep. Int. Whal. Commn. Special Issue* **8**, 252–282 (1986).
16. Parra, G. J., Corkeron, P. J. & Marsh, H. Population sizes, site fidelity and residence patterns of Australian snubfin and Indo-Pacific humpback dolphins: implications for conservation. *Biol. Conserv.* **129**, 167–180 (2006).
17. Nicholson, K., Bejder, L., Allen, S. J., Krützen, M. & Pollock, K. H. Abundance, survival and temporary emigration of bottlenose dolphins (*Tursiops* sp.) off Useless Loop in the western gulf of Shark Bay, Western Australia. *J. Mar. Freshw. Res.* **63**, 1059-1068 (2012).
18. Connor, R. C., Wells, R., Mann, J. & Read, A. The bottlenose dolphin: social relationships in a fission-fusion society in *Cetacean Societies: Field Studies of Whales and Dolphins* (eds. Mann, J., Connor, R., Tyack, P. & Whitehead, H.) 91-126 (University of Chicago Press, 2000).
